# Integrated Genomic, Transcriptomic, and Circulating Biomarkers Predict Benefit to Immune Checkpoint Inhibitor Plus Chemotherapy in Advanced Non‐Small Cell Lung Cancer

**DOI:** 10.1002/mco2.70846

**Published:** 2026-07-06

**Authors:** Lailing Li, Dandan Han, Xiaoliang Zhang, Hui Zhou, Jiajun Li, Tian Tian, Rubing Bai, Ke Xu, Yehong Xu, Cheng He, Linjuan Xu, Hao Wang, Hao Tang, Song Wei, Jun Li, Rui He, Shicheng Niu, Xi Gao, Fufeng Wang, Qifan Jing, Jiani Yin, Ling Xu, Lingling Xu, Zhi‐Hong Zhang

**Affiliations:** ^1^ Department of Respiratory Oncology The First Affiliated Hospital of USTC Division of Life Sciences and Medicine University of Science and Technology of China Hefei China; ^2^ Department of Respiratory Oncology Anhui Provincial Cancer Hospital Hefei China; ^3^ Department of Blood Transfusion The First Affiliated Hospital of USTC Division of Life Sciences and Medicine University of Science and Technology of China Hefei China; ^4^ Department of Blood Transfusion Anhui Provincial Cancer Hospital Hefei China; ^5^ Department of Pathology The First Affiliated Hospital of USTC Division of Life Sciences and Medicine University of Science and Technology of China Hefei China; ^6^ Department of Pathology Anhui Provincial Cancer Hospital Hefei China; ^7^ Bozhou Traditional Chinese Medicine Hospital Anhui Province Bozhou China; ^8^ Department of Pulmonology Taihe County Traditional Chinese Medicine Hospital Fuyang China; ^9^ Nanjing Geneseeq Technology Inc. Nanjing China; ^10^ Department of Respiratory Medicine Anhui Chest Hospital Hefei China; ^11^ Department of Oncology Anhui Chest Hospital Hefei China

**Keywords:** circulating tumor DNA, combination immunotherapy, immune checkpoint inhibitors, multi‐omics, non‐small cell lung cancer, predictive biomarkers

## Abstract

Combined immune checkpoint inhibitor (ICI) and chemotherapy is the standard first‐line treatment for advanced non‐small cell lung cancer (NSCLC) without targetable driver mutations. However, reliable biomarkers predictive of clinical benefit are lacking. We analyzed 54 patients with advanced NSCLC treated first‐line with ICI plus chemotherapy. Pretreatment tumor biopsies underwent targeted DNA and RNA‐seq. Plasma samples for circulating tumor DNA (ctDNA) profiling were collected at multiple timepoints. Associations between genomic, transcriptomic, and ctDNA features and objective response rate (ORR) and progression‐free survival (PFS) were assessed. Genomic analysis revealed that *LRP1B* mutations predicted longer PFS (HR = 0.35, *p* = 0.034), whereas *FBXW7* mutations were associated with shorter PFS (HR = 5.39, *p* = 0.001). Exploratory transcriptomic analysis identified high *SPP1* and *LHCGR* expression as predictors of poor prognosis, while high *IL24* expression correlated with longer PFS. CD8^+^ effector memory T‐cell infiltration was significantly higher in responders (*p* = 0.029). Longitudinal ctDNA analysis showed that positivity at C2D1 (HR = 5.18, *p* = 0.004), C3D1 (HR = 17.81, *p* < 0.001), and C4D1(HR = 3.91, *p* = 0.018) was associated with inferior PFS. This multi‐omics analysis highlights the potential of integrating genomic, transcriptomic, and ctDNA biomarkers to optimize immunotherapy strategies in NSCLC.

## Graphical abstract

This multi‐omics study identified biomarkers associated with clinical benefit from first‐line ICI plus chemotherapy in advanced NSCLC. *LRP1B* mutations and high *IL24* expression correlated with longer progression‐free survival, whereas *FBXW7* mutations, high *SPP1* or *LHCGR* expression, and on‐treatment ctDNA positivity were associated with poor prognosis. These findings highlight the value of integrating genomic, transcriptomic, and dynamic circulating markers to optimize patient stratification.

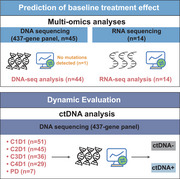

## 1 Introduction

Lung cancer remains the leading cause of cancer‐related morbidity and mortality worldwide, with approximately 2 million new diagnoses and 1.8 million deaths each year [1, 2, 3]. Non‐small‐cell lung cancer (NSCLC) accounts for about 85% of all lung cancer cases. Despite advances in surgery, radiotherapy, chemotherapy, and molecularly targeted agents, the overall prognosis for NSCLC remains poor, with a 5‐year survival rate of approximately 23% [4, 5, 6].

For advanced NSCLC without targetable driver mutations, the combination of immune checkpoint inhibitors (ICIs) and chemotherapy is the standard first‐line option, especially for tumors with programmed death‐ligand 1 (PD‐L1) expression below 50% [7]. While anti‐PD‐1/PD‐L1 agents, such as nivolumab, pembrolizumab, and atezolizumab, have transformed care [8, 9], a substantial proportion of patients derive little or no benefit [10, 11]. Therefore, identifying reliable predictive biomarkers to guide ICI‐based treatment selection and monitoring remains pressing clinical priority.

Current evidence suggests that high PD‐L1 expression and elevated tumor mutational burden (TMB) may predict responsiveness to ICIs in NSCLC [12, 13, 14]. Furthermore, emerging composite biomarkers that capture tumor genomic complexity and immune landscape heterogeneity hold promise for forecasting clinical trajectories [15, 16, 17]. However, their performance is variable, and no single assay reliably predicts benefit across patient populations or treatment combinations. A more integrated view of tumor‐intrinsic features and the tumor–immune microenvironment, coupled with dynamic markers of treatment response, is needed [18, 19, 20, 21].

Recent studies indicate that transcriptomic profiling can capture immune activation, interferon signaling, and myeloid programs linked to ICI sensitivity or resistance [22]. In parallel, liquid biopsy approaches, particularly those based on circulating tumor DNA (ctDNA), offers a minimally invasive approach for early diagnosis, baseline risk stratification, and real‐time response assessment [23, 24]. Longitudinal ctDNA monitoring may offer superior sensitivity compared to conventional imaging in capturing molecular response and emerging resistance. Despite recent studies on NSCLC, the molecular mechanisms of primary and acquired resistance to first‐line ICI plus chemotherapy remain poorly defined, and datasets integrating tumor genomics, transcriptomics, and serial ctDNA data in this setting are particularly limited [10, 11, 17, 25].

To address these gaps, we profiled a prospective cohort of patients with advanced NSCLC treated with ICI plus platinum‐based chemotherapy. Pretreatment tumor biopsies, as well as adjacent immune‐enriched tissues, underwent next‐generation sequencing (NGS) and RNA sequencing (RNA‐seq). In addition, high‐throughput targeted panel sequencing was conducted on longitudinally collected plasma ctDNA samples at each treatment cycle and at disease progression. We assessed associations of genomic alterations, transcriptomic programs, immune‐cell infiltration, and ctDNA dynamics with objective response and progression‐free survival (PFS).

Our objectives were to identify genomic predictors of combination therapy benefit and resistance, to define transcriptomic signatures and immune contexture linked to outcomes, and to evaluate on‐treatment ctDNA kinetics as a dynamic prognostic marker. This integrated multi‐omics framework aims to refine patient selection and monitoring for chemoimmunotherapy in advanced NSCLC and to illuminate biological pathways underpinning response and resistance.

## 2 Results

### 2.1 Baseline Clinical Characteristics and Association With Immunotherapy Response

**FIGURE 1:**
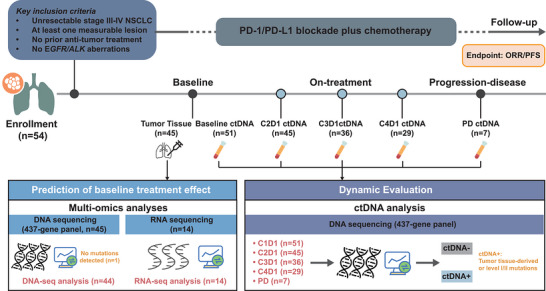
Study design and sample collection. Overview of study timeline, treatment strategy, and biospecimen collection. Baseline tumor tissue was collected for DNA (*n* = 44) and RNA (*n* = 14) sequencing. Plasma samples were collected at five time points (baseline, C2D1, C3D1, C4D1, and progression) for ctDNA analysis (*n* = 51 at baseline). DNA sequencing was performed using a 437‐gene panel. ctDNA positivity was defined by tumor tissue‐derived or level I/II mutations. ORR and PFS were assessed as clinical endpoints. C1D1, cycle 1 day 1; C2D1, cycle 2 day 1; C3D1, cycle 3 day 1; C4D1, cycle 4 day 1; ctDNA, circulating tumor DNA; NSCLC, non‐small cell lung cancer; ORR, objective response rate; PD, progression disease; PFS, progression‐free survival.

This study enrolled 54 patients with unresectable stage III‐IV NSCLC who were treated first‐line with ICIs plus platinum‐based chemotherapy. As illustrated in Figure 1, pretreatment tumor tissue samples underwent targeted DNA sequencing (*n*  =  44) and RNA sequencing (*n* = 14). Plasma samples were collected at baseline (C1D1; *n* = 51), on‐treatment (C2D1 to C4D1), and at disease progression (PD; *n* = 7) for longitudinal ctDNA profiling. Primary endpoints were ORR and PFS.

Baseline clinical characteristics are summarized in Table 1. Median age was 68.5 years (range: 31–80), with 68.5% of patients aged 65 years or older. The majority of patients were male (85.2%). A total of 46.3% of patients had a smoking history, including 3.7% former smokers and 42.6% current smokers. Lung squamous cell carcinoma (LUSC) was the predominant histologic subtype (55.6%), followed by adenocarcinoma (LUAD, 35.2%). At the time of diagnosis, 70.4% of patients presented with stage IV disease, and 29.6% had stage III. PD‐L1 TPS was < 1% in 21.4% of patients, 1–49% in 38.1%, and ≥ 50% in 40.5%. Specific treatment regimens are detailed in Table 1. The most common combination was tislelizumab combined with nab‐paclitaxel and platinum (29.6%). This was followed by sintilimab combined with pemetrexed and platinum (16.7%) and sintilimab combined with nab‐paclitaxel and platinum (16.7%). Additionally, we evaluated the distribution of TMB. Among the evaluable patients, 25 (56.8%) were classified as TMB‐Low and 19 (43.2%) were classified as TMB‐High.

**TABLE 1:** Patient characteristics.

Characteristics	Total (*N* = 54)	LUAD (*N* = 19)	LUSC (*N* = 30)	*p*‐value
Median age, years (range)	68.5 (31–80)	67 (56–78)	68 (31–80)	
**Age group, *n* (%)**				0.539
< 65 years	17 (31.5)	8 (42.1)	9 (30.0)	
≥ 65 years	37 (68.5)	11 (57.9)	21 (70.0)	
**Sex, *n* (%)**				0.665
Female	8 (14.8)	3 (15.8)	3 (10.0)	
Male	46 (85.2)	16 (84.2)	27 (90.0)	
**Smoking status, *n* (%)**				0.695
Never	29 (53.7)	11 (57.9)	14 (46.7)	
Former	2 (3.7)	1 (5.3)	1 (3.3)	
Current	23 (42.6)	7 (36.8)	15 (50.0)	
**Disease stage, *n* (%)**				0.212
IIIA	3 (5.6)	1 (5.3)	2 (6.7)	
IIIB	6 (11.1)	0	6 (20.0)	
IIIC	7 (13.0)	3 (15.8)	4 (13.3)	
IV	38 (70.4)	15 (78.9)	18 (60.0)	
**Histologic type, *n* (%)**				—
LUAD	19 (35.2)	19 (100.0)	0	
LUSC	30 (55.6)	0	30 (100.0)	
PSC	2 (3.7)	0	0	
NSCLC‐NOS	3 (5.6)	0	0	
**PD‐L1% expression in tumor cells, *n* (%)**				0.433
< 1	9 (21.4)	2 (13.3)	6 (27.3)	
1–49	16 (38.1)	8 (53.3)	7 (31.8)	
≥ 50	17 (40.5)	5 (33.3)	9 (40.9)	
Not evaluable	12	4	8	
**TMB, *n* (%)**				0.058
TMB‐L	25 (56.8)	11 (78.6)	14 (46.7)	
TMB‐H	19 (43.2)	3 (21.4)	16 (53.3)	
Not evaluable	10	5	3	
**Treatment regimens, *n* (%)**				
Sintilimab + pemetrexed + platinum	9 (16.7)	9 (47.4)	0 (0.0)	
Sintilimab + nab‐paclitaxel + platinum	9 (16.7)	0	7 (23.3)	
Tislelizumab + pemetrexed + platinum	4 (7.4)	3 (15.8)	0 (0.0)	
Tislelizumab + nab‐paclitaxel + platinum	16 (29.6)	0	16 (53.3)	
Others	16 (29.6)	7 (36.8)	7 (23.3)	

Abbreviations: LUAD, lung adenocarcinoma; LUSC, lung squamous cell carcinoma; nab‐paclitaxel, nanoparticle albumin‐bound paclitaxel; NSCLC‐NOS, non‐small cell lung cancer‐not otherwise specified; PD‐L1, programmed death‐ligand 1; PSC, pulmonary sarcomatoid carcinoma; TMB, tumor mutational burden; TMB‐H, tumor mutational burden‐high; TMB‐L, tumor mutational burden‐low.

According to RECIST criteria, ORR was 64.8% (35/54), and all responses were partial. Stable disease (SD) occurred in 25.9% (14/54) and progressive disease (PD) in 9.3% (5/54) at initial evaluation (Figure S1A). The median follow‐up of the study was 13.7 months (95% CI: 12.5–NA; data cutoff October 24, 2024). Patients with partial response (PR) exhibited significantly longer PFS compared to those with PD or SD (*p* < 0.001; Figure S1D).

PR was more frequently observed in patients with stage III disease compared to stage IV (87.5% vs. 55.3%, *p*  =  0.030; Figure S1C). Other clinical factors, such as age, sex, smoking status, and histologic subtype, were not significantly associated with response (Figure S1B). Univariate Cox regression analysis also showed that disease stage was the only clinical variable significantly associated with PFS (HR  =  3.11, 95% CI: 1.06–9.12, *p*  =  0.030; Figure S1E). Patients with stage III disease showed prolonged PFS than those with stage IV (*p*  =  0.030; Figure S1F).

We also evaluated the predictive value of PD‐L1 expression. Across all TPS categories (< 1%, 1–49%, and ≥ 50%), no significant differences in ORR were observed (Figure S2A,B). Similarly, PD‐L1 expression was not associated with PFS (*p*  =  0.646, Figure S2C). These results suggest limited predictive value of PD‐L1 expression alone in the context of ICI–chemotherapy combination regimens.

### 2.2 Genomic Alterations Associated With Response to Immunotherapy

**FIGURE 2:**
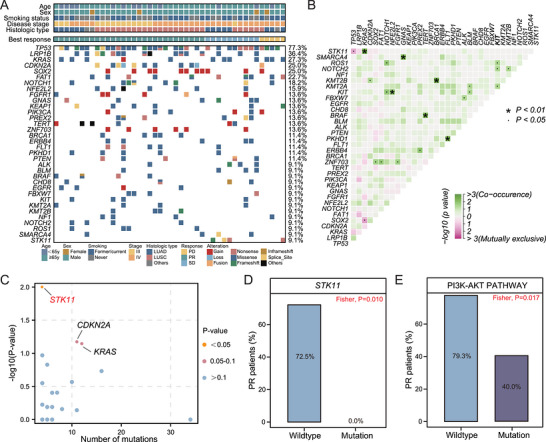
Genomic alterations associated with response to ICI‐based combination therapy. (A) Oncoplot showing the most frequently mutated genes in baseline tumor tissues (*n* = 44). Mutation types and clinical characteristics are annotated. (B) Co‐occurrence and mutual exclusivity matrix of top mutated genes. Green and pink indicate co‐occurring and mutually exclusive gene pairs, respectively. (C) Enrichment analysis identifying genes associated with objective response. *STK11* showed the strongest association with lack of response (*p* < 0.05), followed by *CDKN2A* and *KRAS* (0.05 < *p* < 0.1). (D) Response rates in patients with or without *STK11* mutations. (E) Association between PI3K‐AKT pathway alterations and response rate. ICI, immune checkpoint inhibitor; LUAD, lung adenocarcinoma; LUSC, lung squamous cell carcinoma; PD, progressive disease; PR, partial response; SD, stable disease;.

Building on the clinical associations, we next interrogated tumor‐intrinsic genomics by profiling baseline biopsies with targeted NGS in patients receiving ICI plus chemotherapy. The most frequently mutated genes were *TP53* (77.3%) and *LRP1B* (36.4%), followed by *KRAS* (27.3%), *CDKN2A* (25.0%), *SOX2* (25.0%), and *FAT1* (22.7%) (Figure 2A). Analysis of pairwise relationships revealed patterns of mutual exclusivity and co‐occurrence among recurrent alterations (Figure 2B).

To identify molecular correlates of response, we performed an enrichment analysis associating mutational status with ORR across the cohort (Figure 2C). Among the mutated genes, *STK11* was most strongly associated with lack of response (*p* = 0.010), with trends also observed for *CDKN2A* (*p* = 0.067) and *KRAS* (*p* = 0.071). Consistent with the enrichment results, *STK11*‐mutant tumors had an ORR of 0%, compared to 72.5% in the wild‐type group (*p* = 0.010, Figure 2D). Similarly, patients with *KRAS* and *CDKN2A* mutations exhibited lower response rates compared to wild‐type (*KRAS*: 41.7% vs. 75.0%, *p* = 0.071; *CDKN2A*: 90.9% vs. 57.6%, *p* = 0.067; Figure S3A,B), though the differences were not statistically significant. We also evaluated traditional genomic features such as TMB and CIS, but neither was significantly associated with treatment response in this cohort. Subgroup analyses using multiple stratification approaches confirmed that TMB and CIS levels were not significantly different between responders and non‐responders (Figure S3D,E).

To investigate whether gene‐level associations could be extended to signaling pathways, we assessed the relationship between alterations in major oncogenic pathways and treatment response. PI3K‐AKT signaling was significantly associated with reduced response (*p* = 0.017; Figure S3C). Patients harboring PI3K‐AKT pathway mutations had a lower PR rate compared to those without such alterations (40.0% vs. 79.3%, *p* = 0.017; Figure 2E). This finding is consistent with the involvement of *STK11*, one key component of the PI3K‐AKT pathway, in mediating resistance to immunotherapy [26].

### 2.3 Survival Outcome and Mechanistic Insights of Genomic Alterations in Immunochemotherapy

**FIGURE 3:**
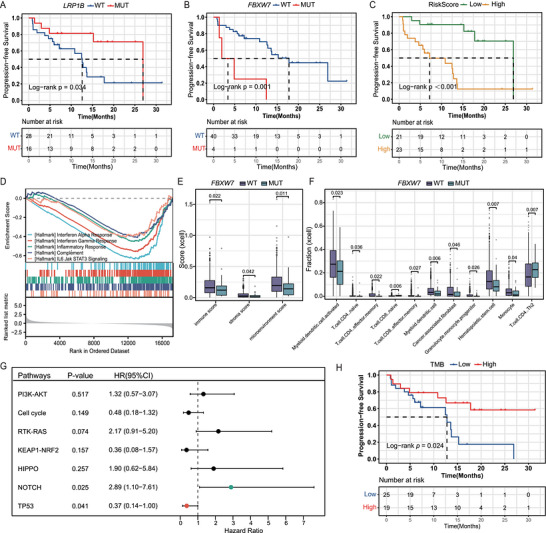
Prognostic significance and mechanistic characterization of genomic alterations in patients receiving ICI‐based combination therapy. (A) Kaplan–Meier curves for PFS according to *LRP1B* mutation status (MUT vs. WT). (B) Kaplan–Meier curves for PFS according to *FBXW7* mutation status (MUT vs. WT). (C) Kaplan–Meier curves for PFS stratified by the composite risk score derived from disease stage, *LRP1B* mutation, and *FBXW7* mutation (high vs. low). (D) GSEA showing significant downregulation of immune‐ and inflammation‐related Hallmark pathways, including interferon α and γ responses, inflammatory response, complement, and IL6–JAK–STAT3 signaling, in *FBXW7*‐mutated tumors. (E) Immune, stroma, and microenvironment scores calculated by xCell in *FBXW7*‐mutated and wild‐type tumors. (F) Relative fractions of selected immune cell subsets in *FBXW7*‐mutated and wild‐type tumors, as estimated by xCell. (G) Forest plot showing hazard ratios for major oncogenic pathways in relation to PFS. NOTCH pathway alterations were associated with worse prognosis, and TP53 pathway alterations were associated with improved prognosis. (H) Kaplan–Meier curves for PFS according to TMB status (high vs. low). HRs and CIs were estimated using univariate Cox proportional hazards models, and *p*‐values were calculated using the log‐rank test. CI, confidence interval; ICI, immune checkpoint inhibitor; GSEA, gene set enrichment analysis; HR, hazard ratio; MUT, mutant; PFS, progression‐free survival; TMB, tumor mutational burden; WT, wild type.

We first performed univariate analyses to evaluate gene‐level association with PFS based on baseline tumor DNA sequencing data. In univariate analyses, *BRCA1* (*p* = 0.018; Figure S4A and Table S1), *KMT2B* (*p* = 0.023; Figure S4B and Table S1), and *LRP1B* (*p* = 0.034; Figure 3A and Table S1) mutations were associated with longer PFS, whereas *FBXW7* (*p* = 0.001; Figure 3B and Table S1) and *KRAS* (*p* = 0.042; Figure S4C and Table S1) mutations were associated with shorter PFS. In a multivariable Cox model integrating genomic and clinical variables, disease stage (*p* = 0.002), *LRP1B* mutation (*p* = 0.009), and *FBXW7* mutation (*p* < 0.001) remained independent predictors of PFS (Table S1). Based on these factors, a composite risk score stratified patients into high‐ and low‐risk groups, with markedly shorter PFS in the high‐risk group (*p* < 0.001; Figure 3C).

To explore mechanisms underpinning these prognostic signals, we performed pathway and transcriptomic analyses focused on *LRP1B* and *FBXW7*. GSEA revealed that *FBXW7*‐mutated tumors exhibited significant downregulation of immune‐ and inflammation‐related pathways, including interferon α and γ responses, inflammatory response, complement, and IL6–JAK–STAT3 signaling (Figure 3D and Table S2). Consistently, xCell deconvolution revealed that *FBXW7*‐mutated tumors had lower immune, stroma, and microenvironment scores compared to wild type (*p* = 0.022, *p* = 0.042, and *p* = 0.011, respectively; Figure 3E). Moreover, multiple immune cell populations, such as activated myeloid dendritic cells, CD4+ and CD8+ naïve and effector memory T cells, cancer‐associated fibroblasts, granulocyte–monocyte progenitors, hematopoietic stem cells, and monocytes, were significantly reduced in the microenvironment of *FBXW7*‐mutated tumors (all *p* < 0.05; Figure 3F).

In contrast, the presence of *LRP1B* mutations was not associated with differences in immune, stroma, or microenvironment scores (all *p* > 0.2; Figure S4B). Instead, *LRP1B*‐mutated tumors were enriched for cell cycle and proliferation‐related pathways, including E2F targets, G2M checkpoint, MYC targets V1 and V2, mitotic spindle, and mTORC1 signaling (Figure S4C and Table S2). Although these pathways are generally associated with tumor proliferation, their activation in the context of immunochemotherapy may reflect a distinct tumor biology associated with better treatment responsiveness. These findings suggest that *FBXW7* mutations may impair antitumor immune responses, whereas *LRP1B* mutations may be linked to proliferative signaling patterns that are associated with improved PFS in this therapeutic setting.

We further evaluated the prognostic relevance of alterations in major oncogenic pathways. Pathway‐level Cox regression analysis identified NOTCH pathway alterations as a significant adverse prognostic factor (*p* = 0.025; HR = 2.89, 95% CI: 1.10–7.61; Figure 3G and Figure S4E) and TP53 pathway alterations as a protective factor (p = 0.041; HR = 0.37, 95% CI: 0.14–1.00; Figure 3G and Figure S4E). Notably, both pathways include genes implicated in the earlier analyses, namely, *LRP1B* and *FBXW7*, providing mechanistic consistency. In addition, high TMB was associated with significantly longer PFS (*p* = 0.024; Figure 3H), whereas CIS showed no significant association (*p* = 0.860; Figure S4F).

### 2.4 Transcriptomic Differences and Immune Landscape Associated With Durable Clinical Benefit

**FIGURE 4:**
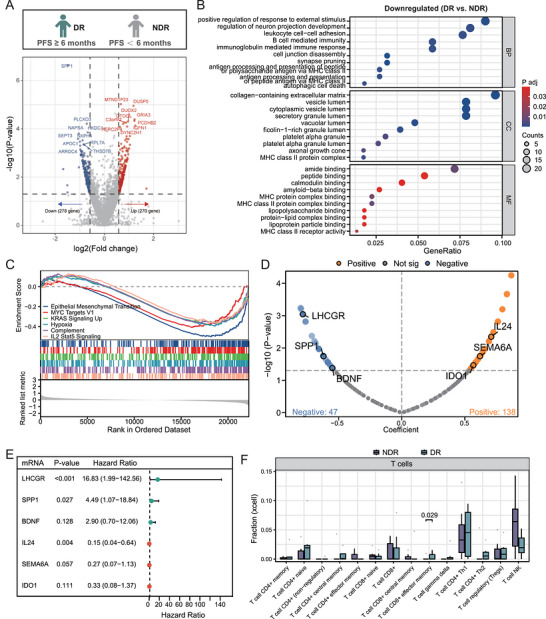
Transcriptomic differences and immune landscape associated with durable clinical benefit in patients receiving ICI‐based combination therapy. (A) Volcano plot showing differentially expressed genes (DEGs) between durable responders (DR, PFS ≥ 6 months) and non‐durable responders (NDR, PFS < 6 months). (B) GO enrichment analysis of genes downregulated in DR versus NDR, highlighting immune‐related processes such as antigen processing and presentation, B‐cell‐mediated immunity, immunoglobulin‐mediated immune responses, leukocyte cell–cell adhesion, and MHC class II–associated functions. (C) GSEA identifies significantly enriched Hallmark pathways in DR versus NDR, with complement and IL2–STAT5 signaling upregulated in DR, and EMT, MYC targets V1, KRAS signaling up, and hypoxia upregulated in NDR. (D) Heatmap of immune‐related DEGs distinguishing DR from NDR, with six candidate genes (*LHCGR, SPP1, BDNF, IL24, SEMA6A*, and *IDO1*) showing significant differential expression. (E) Forest plot of Cox regression analysis showing the association between expression of the six immune‐related DEGs and PFS. High *LHCGR* and *SPP*1 expression were associated with short PFS, whereas high *IL24* expression was associated with prolonged PFS. (F) Relative fractions of immune cell subsets estimated by xCell, showing significantly higher abundance of CD8^+^ effector memory T cells in DR compared with NDR. CI, confidence interval; DEG, differentially expressed gene; DR, durable responder; GO, Gene Ontology; GSEA, gene set enrichment analysis; HR, hazard ratio; ICI, immune checkpoint inhibitor; NDR, non‐durable responder; PFS, progression‐free survival.

To investigate transcriptomic determinants of clinical outcomes, we compared patients with durable response (DR, PFS ≥ 6 months) and non‐durable responders (NDR, PFS < 6 months). A total of 548 DEGs were identified between the two groups (Figure 4A). Genes downregulated in DR versus NDR were enriched for immune‐related processes, including antigen processing and presentation, B‐cell‐mediated immunity, immunoglobulin‐mediated immune responses, leukocyte cell–cell adhesion, and various MHC class II–associated pathways (Figure 4B). Conversely, genes upregulated in DR were enriched for processes related to cellular response to UV‐A, calcium‐dependent cell–cell adhesion, and glutamate receptor signaling (Figure S5A). GSEA further demonstrated that pathways such as epithelial–mesenchymal transition (EMT), MYC targets V1, KRAS signaling up, and hypoxia were significantly enriched in NDR, whereas complement and IL2–STAT5 signaling were positively enriched in DR (Figure 4C). The complete list of enriched pathways from the GSEA is provided in Figure S5B.

Focusing on immune‐related DEGs, we identified six genes (*LHCGR*, *SPP1*, *BDNF*, *IL24*, *SEMA6A*, and *IDO1*) that were differentially expressed between DR and NDR (Figure 4D). Cox regression analysis confirmed that high expression of *LHCGR* (*p* < 0.001, HR = 16.83, 95% CI: 1.99–142.56) and *SPP1* (*p* = 0.027, HR = 4.49, 95% CI: 1.07–18.84) predicted shorter PFS, whereas high *IL24* expression (*p* = 0.004, HR = 0.15, 95% CI: 0.04–0.64) was linked to prolonged PFS. *BDNF* (*p* = 0.128, HR = 2.90), *SEMA6A* (*p* = 0.057, HR = 0.27), and *IDO1* (*p* = 0.111, HR = 0.33) showed consistent but not statistically significant trends (Figure 4E). To assess their predictive performance, receiver operating characteristic (ROC) curve analysis demonstrated strong discrimination of DR versus NDR for all six genes, with area under the curve (AUC) values ranging from 0.816 to 0.949 (Figure S5C).

xCell‐based deconvolution showed no significant differences in immune, stroma, and microenvironment scores between DR and NDR (Figure S5D). However, CD8^+^ effector memory T‐cell infiltration was significantly higher in the DR group (*p* = 0.029; Figure 4F). The complete immune cell infiltration profile for all subsets is shown in Figure S5E.

### 2.5 Dynamic ctDNA Monitoring During Immunochemotherapy and Associations With Recurrence and Survival

**FIGURE 5:**
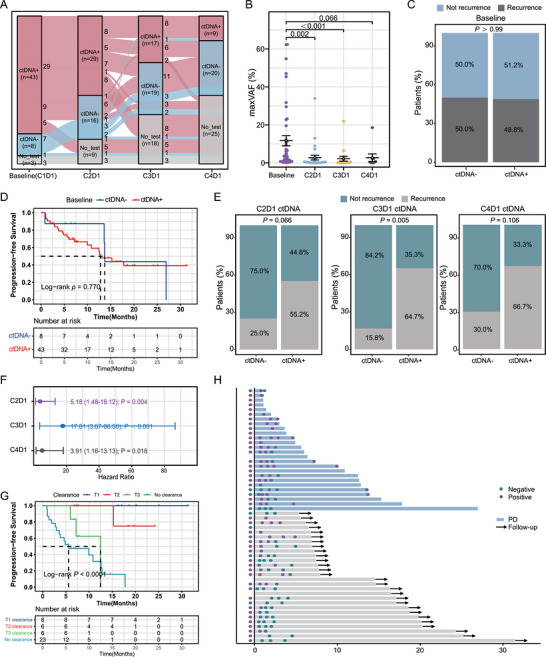
Dynamic changes in ctDNA during immunochemotherapy and their association with recurrence and survival in patients with locally advanced NSCLC. (A) ctDNA positivity rates at baseline (C1D1), after the first cycle (C2D1), after the second cycle (C3D1), and after the third cycle (C4D1) in the entire cohort. (B) Maximum variant allele frequency (maxVAF) across four time points, showing significant declines at C2D1 (*p* = 0.002) and C4D1 (*p* < 0.001) compared with baseline. (C) Recurrence proportions according to baseline ctDNA status, showing no significant difference (*p* > 0.99). (D) Kaplan–Meier curves for progression‐free survival (PFS) according to baseline ctDNA status (*p* = 0.770). (E) Recurrence proportions according to ctDNA status at C2D1 (*p* = 0.066), C3D1 (*p* = 0.106), and C4D1 (*p* = 0.005). (F) Kaplan–Meier curves for PFS according to ctDNA status at C2D1 (*p* = 0.004), C3D1 (*p* < 0.001), and C4D1 (*p* = 0.018). (G) Kaplan–Meier curves for PFS stratified by the earliest on‐treatment time point at which ctDNA became undetectable and remained negative thereafter: T1 clearance (from C2D1 onward), T2 clearance (from C3D1 onward), T3 clearance (from C4D1 onward), and no clearance (*p* < 0.0001). (H) Swimmer plot showing the timing of progressive disease (PD), follow‐up duration, and ctDNA status for each patient. Each horizontal bar represents an individual patient, with blue bars indicating time from treatment initiation to PD and grey bars (with arrows) indicating ongoing follow‐up without PD. Dots denote ctDNA assessment results at different time points: purple for ctDNA‐positive and green for ctDNA‐negative. Patients are ordered by time to PD or last follow‐up. HRs and CIs were estimated using univariate Cox proportional hazards models, and *p*‐values were calculated using the log‐rank test. C1D1, cycle 1 day 1; C2D1, cycle 2 day 1; C3D1, cycle 3 day 1; C4D1, cycle 4 day 1; CI, confidence interval; ctDNA, circulating tumor DNA; HR, hazard ratio; maxVAF, maximum variant allele frequency; PD, progressive disease; PFS, progression‐free survival.

The feasibility of serial plasma ctDNA monitoring and the optimal on‐treatment time point for predicting recurrence were evaluated at four time points: prior to the first (C1D1/baseline), second (C2D1), third (C3D1), and fourth (C4D1) cycles of combination immunotherapy. The ctDNA positivity rates declined over time in response to treatment, with 79.6% (43/54) at C1D1, 53.7% (29/54) at C2D1, 31.5% (17/54) at C3D1, and 16.7% (9/54) at C4D1 (Figure 5A). In patients with matched tumor tissue (*n* = 29), ctDNA positivity rates decreased from 86.2% at C1D1 to 31.0% at C4D1 (Figure S6A). The maximum variant allele frequency (maxVAF) declined significantly at C2D1 (*p* = 0.002) and C4D1 (*p* < 0.001) compared with C1D1, consistent with reduced tumor burden during treatment (Figure 5B).

Recurrence risk increasingly diverged by on‐treatment ctDNA status. At baseline, recurrence proportions were comparable between ctDNA‐negative and ctDNA‐positive patients (*p* > 0.99; Figure 5C). At C2D1, recurrence was more frequent in ctDNA‐positive than ctDNA‐negative patients (55.2% vs. 25.0%, *p* = 0.066; Figure 5E), a pattern persisted at C3D1 (64.7% vs. 15.8%, *p* = 0.106; Figure 5E) and reached significance at C4D1 (66.7% vs. 30.0%, *p* = 0.005; Figure 5E). The sensitivity of ctDNA positivity for predicting recurrence was 0.50, 0.79, and 0.80 at C2D1, C3D1, and C4D1, respectively; specificity was 0.82, 0.73, and 0.48; PPV was 0.67, 0.65, and 0.55; and NPV was 0.70, 0.84, and 0.75, with the highest NPV observed at C3D1 (Figure S6B).

PFS did not differ significantly according to baseline ctDNA status (*p* = 0.770; Figure 5D). However, on‐treatment ctDNA positivity was associated with shorter PFS at C2D1 (*p* = 0.004; Figure S6C), C3D1 (*p* < 0.001; Figure S6D), and C4D1 (*p* = 0.018; Figure S6E). For longitudinal analysis, we further stratified patients by ctDNA clearance according to the earliest on‐treatment time point at which ctDNA became undetectable and remained negative thereafter. T1 clearance referred to ctDNA negativity from C2D1 onward, T2 clearance from C3D1 onward, and T3 clearance from C4D1 onward, while the no‐clearance group remained ctDNA‐positive throughout treatment. PFS showed a stepwise gradient, with the shortest survival outcome in the no‐clearance group, intermediate outcomes in T3 and T2 clearance groups, and the longest survival in the T1 clearance group (*p* < 0.001; Figure 5G).

The timing of PD, follow‐up duration, and ctDNA status for each patient during immunochemotherapy are shown in Figure 5H. ctDNA results at multiple on‐treatment time points were overlaid on individual patient timelines, revealing heterogeneous clearance patterns. Most patients who experienced PD showed persistent or recurrent ctDNA positivity prior to radiographic progression, whereas those with sustained ctDNA negativity throughout follow‐up generally achieved prolonged disease control. A minority showed discordant patterns, with persistent ctDNA negativity despite eventual PD, or cases exhibiting fluctuating ctDNA results during treatment and follow‐up. Overall, these results support that both landmark on‐treatment ctDNA status and early, sustained ctDNA clearance provide important prognostic information in patients receiving immunochemotherapy.

## 3 Discussion

This multi‐omics study investigated genomic, transcriptomic, and ctDNA features associated with clinical outcomes in patients with advanced NSCLC receiving ICI plus chemotherapy. We identified key genomic alterations, immune‐related transcriptional signatures, and dynamic ctDNA patterns that provide mechanistic insights into treatment response and resistance, with potential clinical utility for patient stratification. In our cohort, PD‐L1 TPS and TMB showed limited predictive power for short‐term tumor response, consistent with prior studies indicating that these biomarkers alone are insufficient for optimal patient selection [27, 28, 29]. Higher TMB was associated with longer PFS, suggesting its relevance for long‐term benefit rather than early tumor shrinkage.

PD‐L1 IHC is the first FDA‐approved companion diagnostic for ICIs. However, variability in quantification thresholds, lack of standardized antibody clones, and intratumoral heterogeneity limit its predictive value [27]. In our cohort, PD‐L1 TPS of < 1%, 1–49%, and ≥ 50% were observed in 21.4%, 38.1%, and 40.5% of patients, respectively, consistent with other reports in Chinese populations. Although patients achieving PR tended to have higher median PD‐L1 TPS, no significant correlation was observed between PD‐L1 expression and PFS. These findings highlight the complexity of PD‐L1 as a biomarker, potentially influenced by spatial heterogeneity and microenvironmental factors.

TMB has emerged as another potential biomarker. Consistent with previous clinical trials and real‐world studies [28, 29], our data showed that higher TMB was associated with longer survival. However, TMB was not associated with ORR, suggesting it may be more reflective of long‐term benefit rather than short‐term tumor shrinkage. The limited predictive power of PD‐L1 and TMB emphasizes the need for additional biomarkers that can refine our ability to predict which patients will benefit from ICIs. In this study, we incorporated multidimensional profiling of genomic, transcriptomic, and ctDNA features associated with survival outcomes in patients with NSCLC treated with ICI combination regimens. Understanding the interplay between molecular underpinnings and the immune landscape may help improve strategies for precise immunotherapy.

We next examined the association of tumor genomic characteristics with ORR in our cohort. *STK11* mutations, which are prevalent among Chinese NSCLC patients, were significantly associated with lack of clinical response, consistent with previous findings [30]. Functional pathway analysis revealed enrichment of PI3K‐AKT pathway alterations, including *CDKN2A*, among non‐responders, suggesting a potential role for this pathway in immune resistance [26]. While *KRAS* and *CDKN2A* mutations have been previously linked to poor ICI outcomes [31, 32, 33], no significant associations were observed in our cohort, which may be attributable to the limited sample size. Mechanistic studies have demonstrated the immunosuppressive effects of these alterations. For example, *STK11*‐mutant NSCLC has been reported to exhibit low PD‐L1 expression, reduced ORR, and shorter PFS and OS after ICI‐based therapy [34]. Moreover, concurrent *STK11* and *KRAS* mutations have been associated with lower anti‐tumor activity of ICIs compared with *STK11* wild‐type tumors [35, 36]. *KRAS*‐G12D mutations correlate with reduced PD‐L1 and chemokine (CXCL10/11) expression, fostering an immunosuppressive microenvironment and attenuating ICI sensitivity [37].

Our analysis revealed that *FBXW7* and *KRAS* mutations were associated with shorter PFS, while *BRCA1, KMT2B*, and *LRP1B* mutations predicted longer PFS. In multivariate Cox regression, *FBXW7* emerged as an independent predictor of poor prognosis, whereas *LRP1B* mutation was associated with significantly improved PFS. *FBXW7* encodes an E3 ubiquitin ligase that regulates oncogenic pathways through substrate degradation (e.g., mTOR, MCL‐1, and Snail) [38]. Loss of *FBXW7* function leads to the accumulation of these substrates, particularly Notch, whose aberrant activation has been reported to suppress cytotoxic T‐cell recruitment and function, contributing to an immune‐cold microenvironment with reduced CD8^+^ T‐cell density [39]. In contrast, *LRP1B* is frequently mutated in NSCLC owing to its large genomic size, making it a common passenger mutation in hyper‐mutated tumors and a surrogate marker for elevated TMB [40]. These mutations correlate with increased neoantigen presentation, thereby enhancing tumor immunogenicity and sensitivity to ICI blockade. Previous studies have associated *LRP1B* mutations with increased immune‐cell infiltration and improved survival outcomes in patients receiving ICI plus chemotherapy [41, 42]. However, in our study, although *LRP1B*‐mutated tumors did not show significant differences in immune score, transcriptomic analysis indicated enrichment of cell cycle and proliferative pathways, suggesting a distinct tumor biology potentially favorable for ICI responsiveness. Pathway enrichment analysis further revealed that NOTCH alterations were associated with shorter PFS, while TP53 pathway alterations correlated with longer PFS.

Transcriptomic analysis further provided mechanistic insights into clinical benefit. Durable responders (DR, PFS ≥ 6 months) exhibited downregulation of immune‐related processes such as antigen presentation and B‐cell‐mediated immunity, whereas NDR were enriched for EMT, MYC targets, KRAS signaling, and hypoxia pathways. Among immune‐related differentially expressed genes (DEGs), high *LHCGR* and *SPP1* expression correlated with poor prognosis, while high *IL24* expression was associated with improved PFS. High *SPP1* expression, in particular, has been shown to facilitate the recruitment of tumor‐associated macrophages (TAMs) and promote their polarization toward an immunosuppressive M2 phenotype, creating a barrier to cytotoxic T‐cell function and contributing to ICI resistance [43, 44]. Aligning with prior evidence linking *SPP1* overexpression to poor immunotherapy outcomes, our findings suggest that these transcriptomic features represent immune evasion mechanisms operating parallel to or independently of the PD‐L1 axis. This highlights the value of multi‐omics integration to capture resistance phenotypes that may be missed by PD‐L1 testing alone. Conversely, high expression of *IL24*, a cytokine known to induce tumor apoptosis and promote immune surveillance [45], was associated with improved outcomes, likely by fostering a favorable immune microenvironment for ICI efficacy. Additionally, *LHCGR* overexpression may contribute to chemo‐resistance through activation of proliferation‐related cAMP signaling pathways [46], thereby limiting the efficacy of the combined chemo‐immunotherapy regimen and aligning with the enrichment of EMT and metabolic signatures in NDR. Our immune cell infiltration analysis revealed that CD8^+^ effector memory T cells were significantly enriched in DR, consistent with reports that CD8^+^ T‐cell infiltration predicts benefit from ICI‐based regimens [47, 48, 49].

Longitudinal ctDNA monitoring provided additional prognostic information beyond baseline biomarkers. Early and sustained ctDNA clearance during treatment was strongly predictive of prolonged PFS, consistent with previous studies demonstrating that dynamic ctDNA changes precede radiographic responses [50, 51]. These results reinforce the utility of ctDNA dynamics as a real‐time, non‐invasive biomarker for emerging resistance.

Several limitations of this study should be acknowledged. First, the moderate sample size may limit the statistical power to detect associations involving rare genomic events. To mitigate this limitation, we employed the large‐scale TCGA dataset (*n* = 972) to validate the associations between genomic alterations and immune landscape features, confirming the robustness of our mechanistic findings. In particular, given the limited subset of patients with available RNA‐seq data (*n* = 14), the transcriptomic findings identified in this study should be considered exploratory and hypothesis‐generating. While these signatures provide valuable mechanistic insights, they require validation in larger, independent cohorts to confirm their predictive utility. Second, the relatively short follow‐up duration may lead to underestimation of long‐term survival differences, particularly for patients with ongoing responses at the time of analysis. Although our integrative multi‐omics approach identified associations between *FBXW7* and *LRP1B* mutations, immune modulation, and clinical outcomes, these associations remain correlative. Functional validation in preclinical models and independent patient cohorts is required to clarify the causal relationships. Finally, as this was a single‐center study, external validation in multi‐center settings with larger and more diverse patient populations will be essential to confirm these findings and to refine predictive biomarker models for clinical application.

## 4 Conclusion

This integrative multi‐omics study highlights the limitations of PD‐L1 and TMB as standalone biomarkers for ICI‐based therapy in NSCLC. By combining genomic, transcriptomic, and longitudinal ctDNA data, we identified *FBXW7* mutation as a robust independent predictor of poor prognosis and *LRP1B* mutation as a favorable biomarker. Transcriptomic profiling implicated immune evasion (*SPP1*, low CD8^+^ effector memory T‐cell infiltration) and oncogenic signaling (EMT, KRAS, MYC) as potential drivers of resistance, while dynamic ctDNA clearance provided early prognostic information. These findings support the incorporation of multi‐omics biomarker panels into clinical decision‐making to personalize immunochemotherapy strategies. Although the therapeutic landscape continues to evolve with novel agents such as bispecific antibodies, characterizing fundamental resistance phenotypes like immune exclusion remains essential for optimizing patient selection across current and emerging regimens. Furthermore, the longitudinal ctDNA monitoring framework validated in this study is largely drug‐agnostic, providing a broadly applicable and non‐invasive strategy for early efficacy assessment across different immunotherapy platforms. Limitations include the moderate sample size, which may limit the detection of associations for rare genomic events, and the relatively short follow‐up duration. Functional validation is needed to elucidate the mechanisms by which *FBXW7* and *LRP1B* mutations influence treatment response. Future multi‐center studies with larger cohorts and extended longitudinal profiling will be essential to validate and refine these biomarkers for clinical application.

## 5 Materials and Methods

### 5.1 Patients and Samples

Between March 2022 and May 2024, this prospective cohort study recruited 54 participants diagnosed with advanced NSCLC at The First Affiliated Hospital of University of Science and Technology of China (USTC). All subjects received first‐line therapy consisting of anti‐PD‐1/PD‐L1 inhibitors combined with chemotherapy. The inclusion criteria were defined as follows: (i) age ≥ 18 years, (ii) histologically confirmed advanced NSCLC without sensitizing *EGFR* or *ALK* mutations, (iii) no prior systemic therapy, (iv) an Eastern Cooperative Oncology Group performance status score of 0 to 1, and (v) the presence of at least one measurable lesion as per Response Evaluation Criteria in Solid Tumors (RECIST), version 1.1 [52]. The exclusion criteria included (i) a history of noninfectious pneumonitis requiring glucocorticoids, (ii) active autoimmune disease, and (iii) current systemic immunosuppressive therapy.

Treatment‐naïve tumor samples were obtained and analyzed using the GeneseeqPrime NGS panel (437 cancer‐related genes) and RNA sequencing. Plasma samples were collected at five landmark time points for ctDNA profiling by NGS using the same GeneseeqPrime panel: prior to the first (C1D1), second (C2D1), third (C3D1), fourth (C4D1) cycles of combination immunotherapy, and at disease progression (PD).

This study was approved by the Ethics Committee of The First Affiliated Hospital of USTC (Approval No.: 2022‐16) and conducted in accordance with the Declaration of Helsinki. Informed consent was obtained from all participants.

To extend insights on immune microenvironment beyond our cohort, we also analyzed an external dataset from The Cancer Genome Atlas (TCGA) database. Specifically, we leveraged the TCGA‐Lung Adenocarcinoma (TCGA‐LUAD) and TCGA‐Lung Squamous Cell Carcinoma (LUSC) cohorts (hereafter collectively referred to as TCGA‐NSCLC; *n* = 972) [53] to compare immune composition between *FBXW7‐* or *LRP1B*‐mutated and wild‐type tumors. This complementary analysis enabled evaluation of mutation‐associated immune features in a larger, histology‐diverse population and provided orthogonal support for findings from our transcriptomic profiling.

### 5.2 Assessment of Clinical Outcomes

Treatment response was assessed according to RECIST, version 1.1 [52]. Objective response rate (ORR) was defined as the proportion of patients achieving complete response (CR) or PR. PFS was calculated from the date of the first application of ICI treatment to the date of documented disease progression or death. For patients lost to follow‐up, data were censored on the last date they were known to be progression‐free or alive.

### 5.3 PD‐L1 Expression Analysis

PD‐L1 expression was assessed in formalin‐fixed paraffin‐embedded (FFPE) tumor samples using the Dako PD‐L1 IHC 22C3 pharmDx kit (Agilent Technologies). Expression was quantified as the tumor proportion score (TPS), defined as the percentage of viable tumor cells showing partial or complete membranous PD‐L1 staining.

### 5.4 Sample Processing and DNA Library Construction

Genomic profiling was conducted at Nanjing Geneseeq Technology Inc. (Nanjing, China), a facility accredited by both the College of American Pathologists (CAP) and the Clinical Laboratory Improvement Amendments (CLIA).

For tissue samples, genomic DNA was isolated from de‐paraffinized FFPE sections using QIAamp DNA FFPE Tissue Kit (Qiagen Cat. No. 56404), following the manufacturer's instructions. For liquid biopsy, plasma was separated by centrifugation at 1800 *g* for 10 min, and cell‐free DNA (cfDNA) was extracted using the QIAamp Circulating Nucleic Acid Kit (Qiagen, Cat. No. 55114). To filter out germline variants, genomic DNA from white blood cells served as a matched control, extracted via the DNeasy Blood and Tissue Kit (Qiagen Cat. No. 69504).

DNA concentrations were measured with the Qubit 3.0 dsDNA HS Assay Kit (Life Technologies), while purity and fragment size were verified using a NanoDrop 2000 (Thermo Fisher Scientific) and a Bioanalyzer 2100 (Agilent Technologies), respectively. Sequencing libraries were constructed using the KAPA Hyper Prep Kit (KAPA Biosystems). Targeted enrichment was performed with the GeneseeqPrime panel and the xGen Lockdown Hybridization and Wash Reagents Kit (Integrated DNA Technologies), followed by sequencing on the DNBSEQ‐T7 platform (MGI Tech Co., Ltd.) according to the manufacturer's instructions.

### 5.5 Somatic Genetic Variant Mutation Calling

Raw sequencing data were demultiplexed into FASTQ format via bcl2fastq (V.2.16.0.10) and quality‐trimmed using Trimmomatic [54]. High‐quality reads were mapped to the human genome (hg19, GRCh37) using Burrows‐Wheeler Aligner (BWA‐mem, v0.7.12; https://github.com/lh3/bwa/tree/master/bwakit) [55]. BAM files were generated and sorted using Picard V.1.119, and local realignment around indels and base quality recalibration were performed with the Genome Analysis Toolkit (GATK 3.4.0; https://software.broadinstitute.org/gatk/).

Variant calling for single nucleotide variants (SNVs) and insertions/deletions (indels) was executed using VarScan2. Variants were annotated with ANNOVAR, ClinVar, HGMD, LOVD, 1000 Genomes, ExAC, and gnomAD databases. Variants with a population frequency ≥ 1% in any public or in‐house database were excluded. Only SNVs and indels with a variant allele frequency (VAF) > 1% for tissue and > 0.1% for ctDNA, supported by at least three unique mutant reads, were retained. Matched WBC DNA was used to exclude germline mutations and clonal hematopoiesis.

Copy number variants (CNVs) were identified via CNVkit [56], defining gains as a fold‐change ≥ 1.6 and losses as ≤ 0.6. Gene fusions were detected by DELLY [57], requiring specific split‐read support (tissue ≥ 3; ctDNA ≥ 1). All mutations and fusions were manually validated and verified using the Integrative Genomics Viewer (IGV) [58]. Oncogenic signaling pathway annotation followed the TCGA framework [59]. Tumor mutation burden (TMB) was defined as the total number of somatic mutations (including synonymous mutations) per megabase of the coding region, excluding hotspots and fusions [60]. Patients were classified into TMB‐high (TMB‐H) and TMB‐low (TMB‐L) groups based on a threshold of 10 mut/Mb. The chromosomal instability score (CIS) was defined as the proportion of DNA segments with a log2 ratio > ±0.2 in all the covered regions of the genome [61]. ctDNA positivity was defined as detection of somatic variants that either overlapped with tumor‐derived mutations identified in the matched tissue or corresponded to level I/II oncogenic or likely oncogenic variants as annotated in OncoKB [62].

### 5.6 Preparation of RNA‐Seq Libraries

Total RNA extraction from FFPE tissue was performed using the miRNeasy FFPE Kit (Qiagen, Valencia, CA). The quantity and quality of the total RNA were assessed using NanoDrop 2000 (Thermo Fisher Scientific, Waltham, MA) and an Agilent 2100 Bioanalyzer (Agilent Technologies, Santa Clara, CA), respectively. Libraries were prepared from extracted RNA using the KAPA Stranded mRNA‐Seq Kit (KAPA Biosystems, Wilmington, MA), per the manufacturer's instructions using 1 µg of RNA with at least an RNA integrity number of 7. The pooled libraries were paired‐end sequenced on the DNBSEQ‐T7 platform.

### 5.7 RNA Sequencing Data Analysis

RNA‐seq data in FASTQ files underwent initial demultiplexing and quality control using Trimmomatic [54]. BWA was then used to remove transfer RNA and ribosomal RNA reads. Adaptor and primer sequences, as well as short reads less than 20 bp, were also excluded. Transcriptomic mapping was executed through STAR software (v2.7.3a) [63], aligning the data to the reference human genome (hg19). Gene‐level quantification was performed using RSEM (version 1.2.31) [64], and gene expression was quantified as transcripts per million (TPM).

Patients were stratified into high and low‐expression groups according to the median TPM value of genes. DEGs were analyzed using the R package “DESeq2.” The thresholds for identifying DEGs were log2 (fold change) > ± 1.5, and *p*‐value < 0.05. Correlation analysis was used to identify immune‐related DEGs that were highly correlated with the response groups. Pathway enrichment analysis of Gene Ontology (GO) and Gene Set Enrichment Analysis (GSEA) was carried out using the R package “ClusterProfiler.”

Immune cell infiltration was estimated using xCELL [65] from TIMER2.0 website (http://timer.cistrome.org/), which provides immune and stromal scores reflecting cell composition within the tumor microenvironment, inversely correlating with tumor purity.

### 5.8 Statistical Analysis

All statistical analyses were performed in R (version 4.1.3). Fisher's exact test was used for categorical variables, and the Wilcoxon rank‐sum test was applied for continuous variables. Kaplan–Meier analysis with log‐rank testing was used for survival comparisons. Hazard ratios (HR) with 95% confidence intervals (CI) were calculated using Cox proportional hazards model. A two‐sided *p* value of less than 0.05 was considered significant for all tests unless indicated otherwise.

## Author Contributions

**Lailing Li**: formal analysis, funding acquisition, writing – original draft. **Dandan Han**: formal analysis, writing – original draft. **Xiaoliang Zhang**: formal analysis, writing – original draft. **Hui Zhou**: investigation. **Jiajun Li**: investigation. **Tian Tian**: investigation. **Rubing Bai**: investigation. **Ke Xu**: investigation. **Yehong Xu**: investigation. **Cheng He**: investigation. **Linjuan Xu**: investigation. **Hao Wang**: investigation. **Hao Tang**: investigation. **Song Wei**: investigation. **Jun Li**: investigation. **Rui He**: investigation. **Shicheng Niu**: investigation. **Xi Gao**: investigation. **Fufeng Wang**: investigation. **Qifan Jing**: investigation. **Jiani Yin**: investigation. **Ling Xu**: conceptualization, methodology, supervision, writing – review and editing. **Lingling Xu**: conceptualization, methodology, supervision, writing – review and editing. **Zhi‐Hong Zhang**: conceptualization, methodology, supervision, writing – review and editing. All authors have read and approved the final manuscript.

## Funding

This work was supported by the Qinglan Project of Anhui Provincial Cancer Hospital (grant number QLGC2024012) to Lailing Li.

## Ethic Statement

The study was approved by the Ethics Committee of The First Affiliated Hospital of USTC (Approval No.: 2022‐16). Written informed consent was obtained from each patient before sample collection.

## Consent

All patients provided written informed consent agreeing to the publication of their data.

## Conflicts of Interest

Fufeng Wang, Qifan Jing, and Jiani Yin are employees of Nanjing Geneseeq Technology Inc, but have no potential relevant financial or non‐financial interests to disclose. The other authors have no conflicts of interest to declare.

## Supporting information




**Supporting Figure 1**: Baseline clinical characteristics and their association with response and PFS in patients receiving ICI plus chemotherapy.
**Supporting Figure 2**: PD‐L1 tumor proportion score (TPS) distribution and its association with response and PFS in patients receiving ICI plus chemotherapy.
**Supporting Figure 3**: Associations of specific gene mutations, oncogenic pathways, TMB, and CIS with treatment response in patients receiving ICI plus chemotherapy.
**Supporting Figure 4**: Associations of specific gene mutations, immune landscape, and oncogenic pathways with survival outcomes in patients receiving ICI plus chemotherapy.
**Supporting Figure 5**: Transcriptomic differences and immune landscape associated with response to ICI plus chemotherapy.
**Supporting Figure 6**: Longitudinal ctDNA dynamics in matched tumor–plasma pairs and their prognostic significance during immunochemotherapy.
**Supporting Table 1**: Univariate and multivariate Cox regression analyses of clinical and genomic factors associated with progression‐free survival.
**Supporting Table 2**: Gene set enrichment analysis (GSEA) of hallmark pathways associated with *FBXW7* and *LRP1B* mutations in the TCGA‐NSCLC cohort.

## Data Availability

The datasets generated and analyzed during this current study are available from the corresponding author upon reasonable request.
