# Supplementary material for: Integrated Genomic, Transcriptomic, and Circulating Biomarkers Predict Benefit to Immune Checkpoint Inhibitor Plus Chemotherapy in Advanced Non‐Small Cell Lung Cancer
Source: MedComm (2020). 2026 Jul 6;7(7):e70846. doi: 10.1002/mco2.70846 (PMC13338630; doi:10.1002/mco2.70846)
Supplement: Supplementary file 1 — Supporting Figure 1: Baseline clinical characteristics and their association with response and PFS in patients receiving ICI plus chemotherapy. Supporting Figure 2: PD‐L1 tumor proportion score (TPS) distribution and its association with response and PFS in patients receiving ICI plus chemotherapy. Supporting Figure 3: Associations of specific gene mutations, oncogenic pathways, TMB, and CIS with treatment response in patients receiving ICI plus chemotherapy. Supporting Figure 4: Associations of specific gene mutations, immune landscape, and oncogenic pathways with survival outcomes in patients receiving ICI plus chemotherapy. Supporting Figure 5: Transcriptomic differences and immune landscape associated with response to ICI plus chemotherapy. Supporting Figure 6: Longitudinal ctDNA dynamics in matched tumor–plasma pairs and their prognostic significance during immunochemotherapy. Supporting Table 1: Univariate and multivariate Cox regression analyses of clinical and genomic factors associated with progression‐free survival. Supporting Table 2: Gene set enrichment analysis (GSEA) of hallmark pathways associated with FBXW7 and LRP1B mutations in the TCGA‐NSCLC cohort. [file MCO2-7-e70846-s001.docx]

**Integrated Genomic, Transcriptomic, and Circulating Biomarkers Predict Benefit to** **Immune Checkpoint Inhibitor plus Chemotherapy in Advanced Non-Small Cell Lung Cancer**

Lailing Li^#1^, Dandan Han^#2^, Xiaoliang Zhang^#3^, Hui Zhou^1^, Jiajun Li^1^, Tian Tian^1^, Rubing Bai^1^, Ke Xu^1^, Yehong Xu^1^, Cheng He^1^, Linjuan Xu^1^, Hao Wang^1^, Hao Tang^1^, Song Wei^1^, Jun Li^1^, Rui He^4^, Shicheng Niu^5^, Xi Gao^4^, Fufeng Wang^6^, Qifan Jing^6^, Jiani Yin^6^, Ling Xu^*7^, Lingling Xu^*8^, Zhi-Hong Zhang^*1^,

^1^Department of Respiratory Oncology, The First Affiliated Hospital of USTC, Division of Life Sciences and Medicine, University of Science and Technology of China, Hefei 230031, China.

^2^Department of Blood Transfusion, The First Affiliated Hospital of USTC, Division of Life Sciences and Medicine, University of Science and Technology of China, Hefei 230031, China.

^3^Department of Pathology, The First Affiliated Hospital of USTC, Division of Life Sciences and Medicine, University of Science and Technology of China, Hefei 230031, China.

^4^Bozhou Traditional Chinese Medicine Hospital Anhui Province, Bozhou 236800, China.

^5^Department of Pulmonology, Taihe County Traditional Chinese Medicine Hospital, Fuyang 236000, China.

^6^Nanjing Geneseeq Technology Inc., Nanjing 210000, China.

^7^Department of Respiratory Medicine, Anhui Chest Hospital, Hefei 230031, China.

^8^Department of Oncology, Anhui Chest Hospital, Hefei 230031, China.

^#^ These authors contributed equally to this work. *Corresponding Authors

Correspondence to:

Professor Zhi-Hong Zhang, Department of Respiratory Oncology, The First Affiliated Hospital of USTC, Division of Life Sciences and Medicine, University of Science and Technology of China, 107 Huanhu East Road, Hefei 230031, China. Email: zhangzhihope2024@163.com

Professor Ling Xu, Department of Respiratory Medicine, Anhui Chest Hospital, 392 Jixi Road, Xuanwu District, Hefei 230031, China. Email: xuling810628@126.com

Professor Lingling Xu, Department of Respiratory Medicine, Anhui Chest Hospital, 392 Jixi Road, Xuanwu District, Hefei 230031, China. Email: xll6338@163.com

**Supplementary Figures**

**
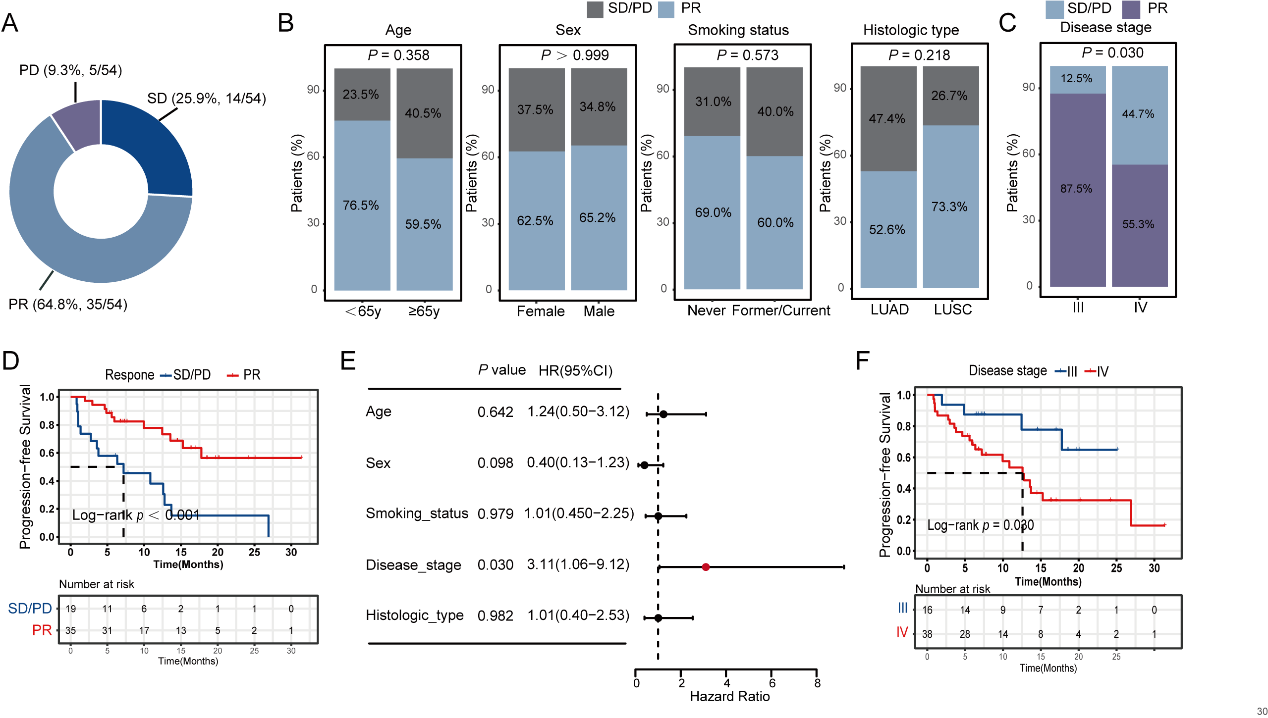
**

**Figure S1.** Baseline clinical characteristics and their association with response and PFS in patients receiving ICI plus chemotherapy.

A. Best overall response distribution per RECIST: partial response (PR) 64.8% (35/54), stable disease (SD) 25.9% (14/54), and progressive disease (PD) 9.3% (5/54).

B. Proportions of PR versus SD/PD across baseline subgroups: age (<65 vs. ≥65 years, P = 0.358), sex (female vs. male, P > 0.999), smoking status (never vs. former/current, P = 0.573), and histologic type (LUAD vs. LUSC, P = 0.218).

C. Proportions of PR versus SD/PD by disease stage (III vs. IV, P = 0.030).

D. Kaplan–Meier curves for progression-free survival (PFS) according to best response (PR vs. SD/PD; log-rank P < 0.001).

E. Univariate Cox regression for PFS with clinical covariates: age (HR = 1.24, 95% CI: 0.50–3.12; P = 0.642), sex (HR = 0.40, 95% CI: 0.13–1.23; P = 0.098), smoking status (HR = 1.01, 95% CI: 0.45–2.25; P = 0.979), disease stage (HR = 3.11, 95% CI: 1.06–9.12; P = 0.030), and histologic type (HR = 1.01, 95% CI: 0.40–2.53; P = 0.982).

F. Kaplan–Meier curves for PFS by disease stage (III vs. IV; log-rank P = 0.030).

Abbreviations: PR, partial response; SD, stable disease; PD, progressive disease; LUAD, lung adenocarcinoma; LUSC, lung squamous cell carcinoma; PFS, progression-free survival; HR, hazard ratio; CI, confidence interval.


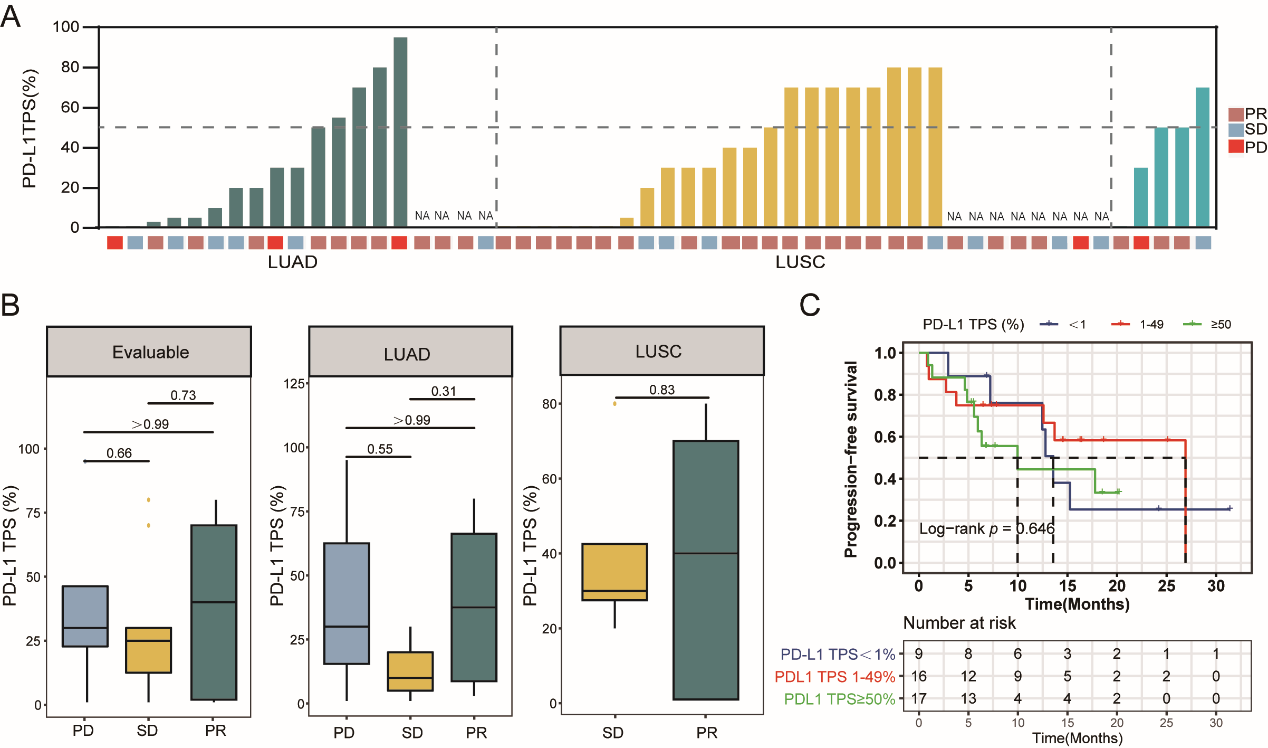


**Figure S2**. PD-L1 tumor proportion score (TPS) distribution and its association with response and PFS in patients receiving ICI plus chemotherapy.

A. Distribution of PD-L1 TPS in patients with lung adenocarcinoma (LUAD) and lung squamous cell carcinoma (LUSC), color-coded by best overall response: partial response (PR), stable disease (SD), or progressive disease (PD). “NA” indicates missing PD-L1 data.

B. Comparison of PD-L1 TPS among PD, SD, and PR groups in the overall evaluable cohort, LUAD subgroup, and LUSC subgroup (all P > 0.05).

C. Kaplan–Meier curves for progression-free survival (PFS) according to PD-L1 TPS categories: <1%, 1–49%, and ≥50% (P = 0.646).

Abbreviations: LUAD, lung adenocarcinoma; LUSC, lung squamous cell carcinoma; PD-L1, programmed death-ligand 1; TPS, tumor proportion score; PR, partial response; SD, stable disease; PD, progressive disease; PFS, progression-free survival.


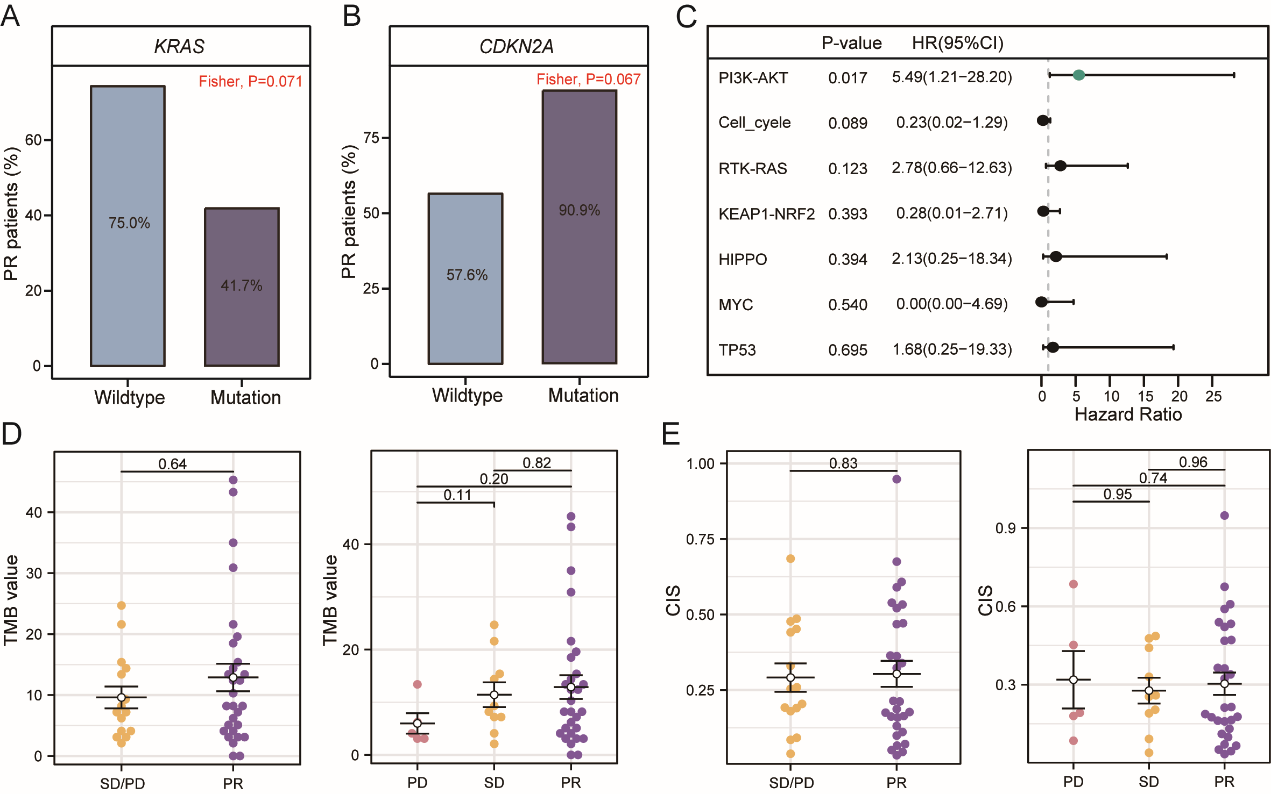


**Figure S3.** Associations of specific gene mutations, oncogenic pathways, TMB, and CIS with treatment response in patients receiving ICI plus chemotherapy.

A. Proportion of partial response (PR) in patients with wild-type versus mutant *KRAS* (75.0% vs. 41.7%; P = 0.071).

B. Proportion of PR in patients with wild-type versus mutant *CDKN2A* (57.6% vs. 90.9%; = P = 0.067).

C. Univariate Cox regression analysis for progression-free survival (PFS) by major oncogenic pathways, showing significant association of PI3K–AKT pathway alterations with shorter PFS (HR = 5.49, 95% CI: 1.21–28.20; P = 0.017).

D. Tumor mutation burden (TMB) values in patients with SD/PD versus PR (left) and in PD, SD, and PR subgroups (right), with no statistically significant differences (all P > 0.05).

E. Chromosomal instability score (CIS) values in patients with SD/PD versus PR (left) and in PD, SD, and PR subgroups (right), with no statistically significant differences (all P > 0.05).

Abbreviations: PR, partial response; SD, stable disease; PD, progressive disease; HR, hazard ratio; CI, confidence interval; TMB, tumor mutation burden; CIS, chromosomal instability score; PFS, progression-free survival; ICI, immune checkpoint inhibitor.


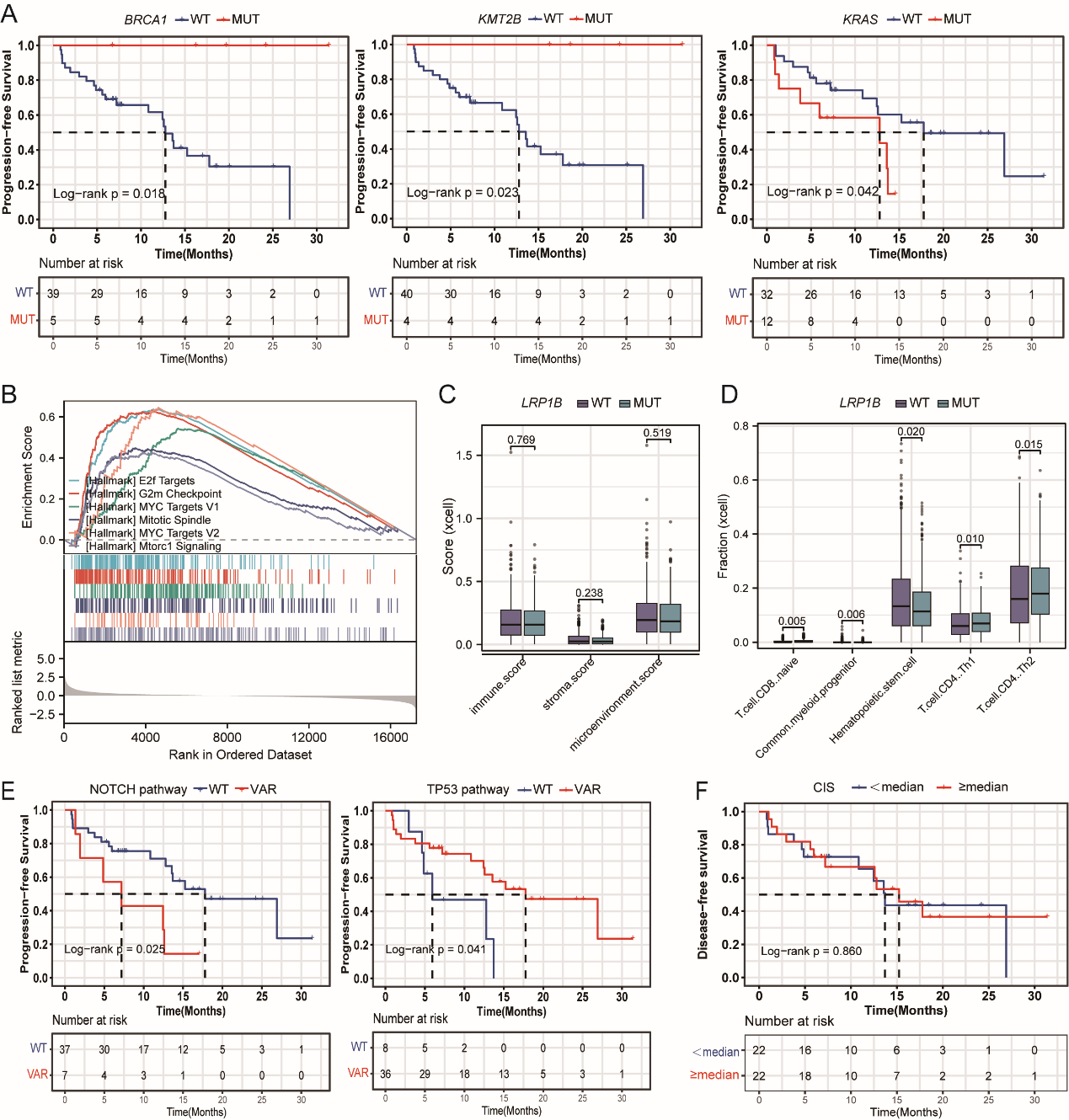


**Figure S4.** Associations of specific gene mutations, immune landscape, and oncogenic pathways with survival outcomes in patients receiving ICI plus chemotherapy.

A. Kaplan–Meier curves for progression-free survival (PFS) according to *BRCA1, KMT2B,* and *KRAS* mutation status (log-rank P = 0.018, 0.023, and 0.042, respectively).

B. Gene set enrichment analysis (GSEA) in the TCGA-NSCLC cohort showing that *LRP1B*-mutated tumors are enriched for cell cycle–related Hallmark gene sets (E2F targets, G2M checkpoint, MYC targets V1/V2, and mitotic spindle).

C. Immune, stroma, and microenvironment scores calculated by xCell in *LRP1B*-mutated versus wild-type tumors, showing no significant differences (all P > 0.05).

D. Relative fractions of selected immune cell subsets in LRP1B-mutated versus wild-type tumors, showing significantly higher infiltration of T cells CD8+ naïve (P = 0.005), common myeloid progenitors (P = 0.020), hematopoietic stem cells (P = 0.010), T cells CD4+ Th1 (P = 0.015), and T cells CD4+ Th2 (P = 0.015) in mutant tumors.

E. Kaplan–Meier curves for PFS according to NOTCH pathway (left) and TP53 pathway (right) alteration status (log-rank P = 0.024 and 0.041, respectively).

F. Kaplan–Meier curve for PFS according to chromosomal instability score (CIS) (< median vs. ≥ median; log-rank P = 0.860).

Abbreviations: WT, wild type; MUT, mutation; VAR, variant; HR, hazard ratio; CI, confidence interval; GSEA, gene set enrichment analysis; CIS, chromosomal instability score; PFS, progression-free survival; ICI, immune checkpoint inhibitor; TCGA, The Cancer Genome Atlas.


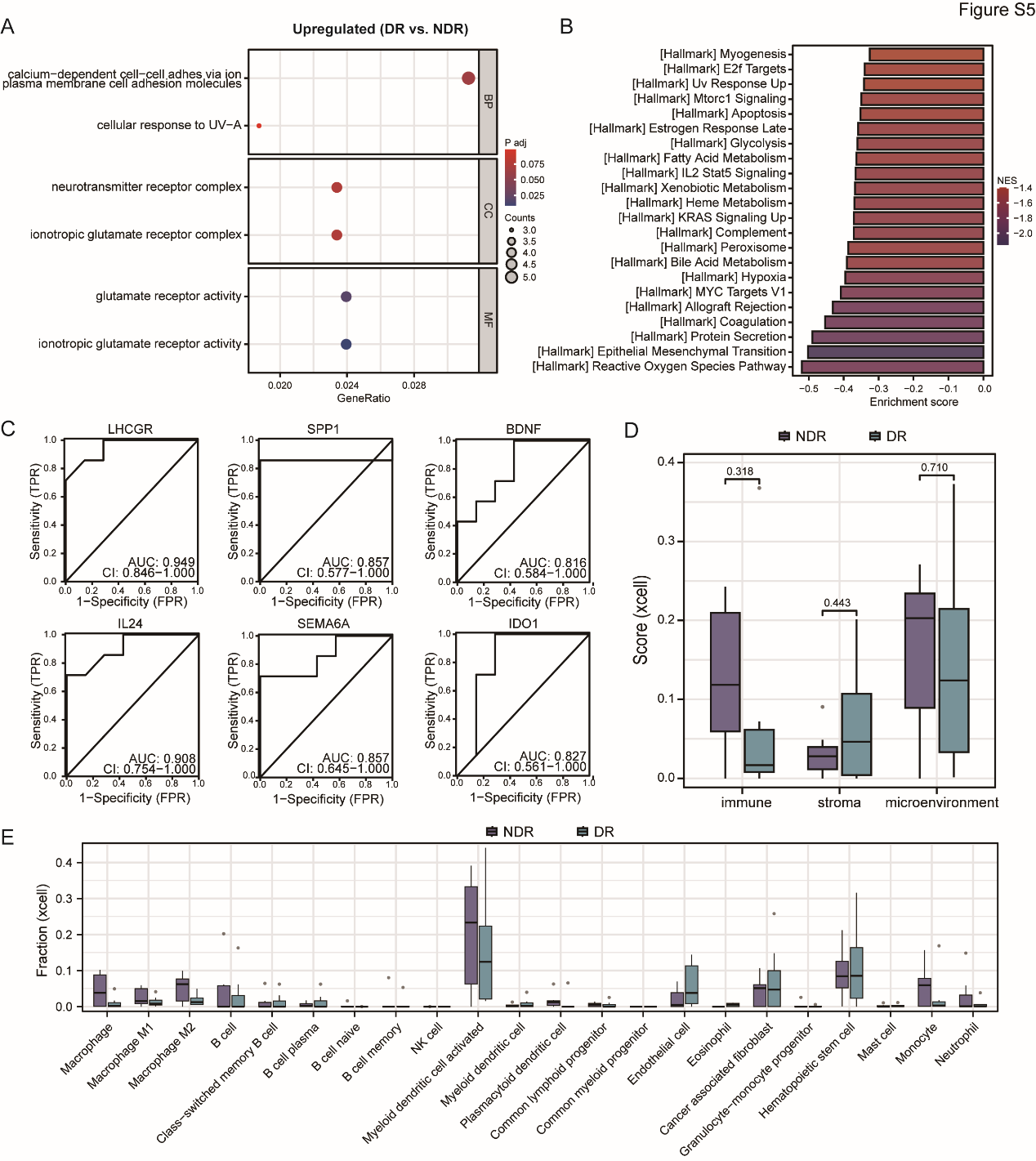


**Figure S5**. Transcriptomic differences and immune landscape associated with response to ICI plus chemotherapy.

A. GO enrichment analysis of genes upregulated in durable responders (DR) versus non-durable responders (NDR), highlighting immune-related processes such as calcium-dependent cell–cell adhesion, plasma membrane cell adhesion molecules, and neurotransmitter receptor complex.

B. GSEA identifying significantly enriched Hallmark pathways in DR versus NDR. Pathways such as E2F targets, KRAS signaling up, complement, IL2–STAT5 signaling, and hypoxia were differentially regulated between the groups.

C. Receiver operating characteristic (ROC) curves for six candidate genes (*LHCGR, SPP1, BDNF, IL24, SEMA6A,* and *IDO1*) distinguishing DR from NDR, with corresponding AUC values and 95% confidence intervals.

D. Immune, stroma, and microenvironment scores calculated by xCell in DR and NDR groups, showing no significant differences (all P > 0.05).

E. Relative fractions of selected immune cell subsets in DR and NDR groups as estimated by xCell, showing differences in macrophage, B cell, dendritic cell, progenitor cell, and granulocyte subsets.

Abbreviations: DR, durable responder; NR, non-durable responder; GO, Gene Ontology; GSEA, gene set enrichment analysis; ROC, receiver operating characteristic; AUC, area under the curve; xCell, cell type enrichment analysis tool; ICI, immune checkpoint inhibitor.


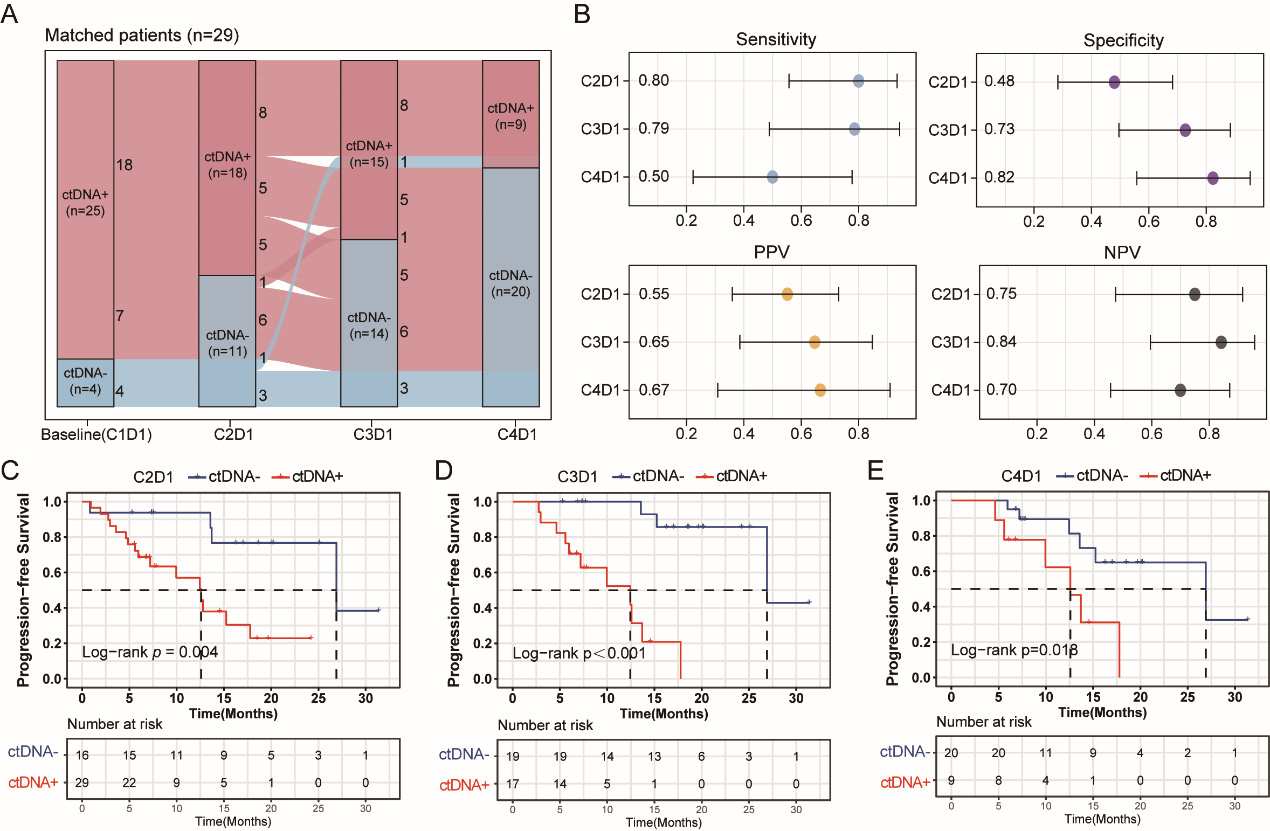


**Figure S6.** Longitudinal ctDNA dynamics in matched tumor–plasma pairs and their prognostic significance during immunochemotherapy.

A. Sankey diagram showing serial ctDNA status transitions from baseline (C1D1) to C2D1, C3D1, and C4D1 in 29 patients with matched tumor tissue sequencing. Blue indicates ctDNA-negative and red indicates ctDNA-positive status.

B. Sensitivity, specificity, positive predictive value (PPV), and negative predictive value (NPV) of ctDNA positivity at C2D1, C3D1, and C4D1 for predicting disease recurrence.

C–E. Kaplan–Meier curves for progression-free survival (PFS) according to ctDNA status at C2D1 (C), C3D1 (D), and C4D1 (E), showing significantly shorter PFS in ctDNA-positive patients at each time point.

Abbreviations: ctDNA, circulating tumor DNA; PPV, positive predictive value; NPV, negative predictive value; PFS, progression-free survival; C1D1, cycle 1 day 1; C2D1, cycle 2 day 1; C3D1, cycle 3 day 1; C4D1, cycle 4 day 1.

**Supplementary Tables**

| **Table S1.** Univariate and multivariate Cox regression analyses of clinical and genomic factors associated with progression-free survival. | | | | |
| --- | --- | --- | --- | --- |
| Factors | Univariate analysis | | Multivariate analysis | |
|  | HR(95% CI) | P-value | HR(95% CI) | P-value |
| Disease stage (IV vs. III) | 3.29(1.10-9.90) | 0.025 | 29.15(3.35-253.64) | 0.002 |
| *BRCA1* (MUT vs. WT) | 0.00(0.00-Inf) | 0.018 | 0.00(0.00-Inf) | ＞0.999 |
| *FBXW7* (MUT vs. WT) | 5.39(1.69-17.23) | 0.001 | 59.34(7.08-497.58) | ＜0.001 |
| *KMT2B* (MUT vs. WT) | 0.00(0.00-Inf) | 0.023 | 0.00(0.00-Inf) | ＞0.999 |
| *KRAS* (MUT vs. WT) | 2.50(1.00-6.24) | 0.042 | 0.77(0.26-2.27) | 0.629 |
| *LRP1B* (MUT vs. WT) | 0.35(0.13-0.96) | 0.034 | 0.12(0.02-0.58) | 0.009 |
| ***Abbreviations***: CI, confidence interval; HR, hazard ratio; Inf, infinity; MUT, mutant; WT, wild type. | | | | |

| **Table S2**. Gene set enrichment analysis (GSEA) of hallmark pathways associated with *FBXW7* and *LRP1B* mutations in the TCGA-NSCLC cohort. | | | | | | |
| --- | --- | --- | --- | --- | --- | --- |
| **Pathway** | **setSize** | **enrichmentScore** | **NES** | **pvalue** | **p.adjust** | **qvalue** |
| **Enriched in FBXW7 Mutant vs. Wild-type** |  |  |  |  |  |  |
| HALLMARK_E2F_TARGETS | 186 | 0.636801 | 2.618028 | 1E-10 | 1E-09 | 6.53E-10 |
| HALLMARK_G2M_CHECKPOINT | 184 | 0.631865 | 2.595944 | 1E-10 | 1E-09 | 6.53E-10 |
| HALLMARK_INTERFERON_ALPHA_RESPONSE | 92 | -0.63626 | -2.26768 | 1E-10 | 1E-09 | 6.53E-10 |
| HALLMARK_INTERFERON_GAMMA_RESPONSE | 196 | -0.55813 | -2.20205 | 1E-10 | 1E-09 | 6.53E-10 |
| HALLMARK_ALLOGRAFT_REJECTION | 195 | -0.54418 | -2.14569 | 1E-10 | 1E-09 | 6.53E-10 |
| HALLMARK_MYC_TARGETS_V1 | 187 | 0.543674 | 2.236947 | 7.78E-10 | 6.49E-09 | 4.23E-09 |
| HALLMARK_INFLAMMATORY_RESPONSE | 197 | -0.45145 | -1.78534 | 4.21E-06 | 3.01E-05 | 1.96E-05 |
| HALLMARK_MITOTIC_SPINDLE | 196 | 0.447978 | 1.864091 | 7.3E-06 | 4.57E-05 | 2.98E-05 |
| HALLMARK_MYC_TARGETS_V2 | 57 | 0.645135 | 2.140965 | 1.49E-05 | 8.29E-05 | 5.41E-05 |
| HALLMARK_KRAS_SIGNALING_UP | 193 | -0.43685 | -1.72587 | 1.75E-05 | 8.76E-05 | 5.72E-05 |
| HALLMARK_MTORC1_SIGNALING | 192 | 0.425138 | 1.754038 | 5.33E-05 | 0.000242 | 0.000158 |
| HALLMARK_COAGULATION | 135 | -0.46459 | -1.74864 | 0.00011 | 0.000458 | 0.000299 |
| HALLMARK_KRAS_SIGNALING_DN | 186 | 0.41771 | 1.717298 | 0.000274 | 0.001052 | 0.000687 |
| HALLMARK_COMPLEMENT | 195 | -0.41122 | -1.6214 | 0.000314 | 0.00112 | 0.000731 |
| HALLMARK_IL6_JAK_STAT3_SIGNALING | 87 | -0.40851 | -1.4285 | 0.012841 | 0.042805 | 0.027936 |
| **Enriched in *LRP1B* Mutant vs. Wild-type** |  |  |  |  |  |  |
| HALLMARK_E2F_TARGETS | 186 | 0.698438 | 2.746273 | 1E-10 | 2.5E-09 | 2E-09 |
| HALLMARK_G2M_CHECKPOINT | 184 | 0.658453 | 2.57795 | 1E-10 | 2.5E-09 | 2E-09 |
| HALLMARK_MITOTIC_SPINDLE | 196 | 0.444627 | 1.72851 | 4.99E-05 | 0.000831 | 0.000665 |
| HALLMARK_MYC_TARGETS_V1 | 187 | 0.417374 | 1.631742 | 0.000418 | 0.005225 | 0.00418 |
| ***Abbreviations***: Adj. P-value, adjusted P-value (Benjamini-Hochberg method); GSEA, gene set enrichment analysis; NES, normalized enrichment score. | | | | | | |
| ***Note:*** Positive NES values indicate pathways relatively enriched in mutant tumors compared to wild-type tumors, whereas negative NES values indicate pathways relatively downregulated in mutant tumors. | | | | | | |
